# Supplementary material for: The Evolution and Characterization of the RNA Interference Pathways in Lophotrochozoa
Source: Genome Biol Evol. 2024 May 7;16(5):evae098. doi: 10.1093/gbe/evae098 (PMC11114477; doi:10.1093/gbe/evae098)
Supplement: evae098_Supplementary_Data [file evae098_supplementary_data.zip › Supplementary_materials.zip!/selected_miRNAs/NoGe_selected_miRNAs/mirdeep_pdf_files/NMRB01000076.1_Notospermus_geniculatus_strain_Ushimado_scaffold76,_whole_genome_shotgun_sequence_2797.pdf]

Provisional ID	:	NMRB01000076.1_Notospermus_geniculatus_strain_Ushimado_scaffold76,_whole_genome_shotgun_sequence_2797
Score total	:	131.6
Score for star read(s)	:	3.9
Score for read counts	:	126.6
Score for mfe	:	1.8
Score for randfold	:	0
Score for cons. seed	:	-0.6
Total read count	:	260
Mature read count	:	199
Loop read count	:	0
Star read count	:	61

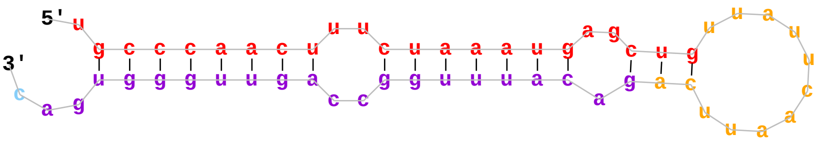

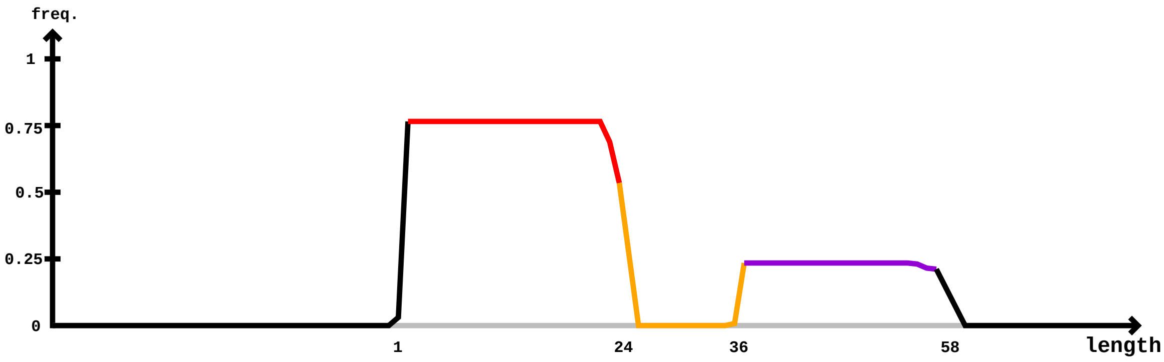


```
                                        Mature                            Star
5'- aucggaguuuccaaagaacauugguccuggauuguugcccaacuuucuaaaugagcuguuauucaauucagacauuuggccaguuggguga cuguuucagguugagcucca  -3'   obs
    aucggaguuuccaaagaacauugguccuggauuguugcccaacuuucuaaaugagcuguuauucaauucagacauuuggccaguugggugacuguuucagguugagcucca        exp
    ...(((((((((((......)))).((((((..(((((((((((..(((((((..(((..........))).)))))))..)))))))))))...))))))..))))))).   reads     mm     sample
    ..................................uugcccaacuuucuaaaugagc.......................................................   1         0      Fm3
    ......................................................................gacauuuggccaguugggu......................   3         0      Fm3

    ......................................................................gacauuuggccaguugggu......................   1         0      Fm1
    ......................................................................gacauuuggccaguuggguga....................   6         0      Fm1
    ......................................................................gacauuuggccaguugggugG....................   1         1      Fm1
    ......................................................................gacauuuggccaguugggugacu..................   2         0      Fm1

    ..................................uugcccaacuuucuaaaCgagc.......................................................   1         1      Mm2
    ..................................uuAcccaacuuucuaaaugagcu......................................................   1         1      Mm2
    ..................................uugcccaacuuucuaaaugagcu......................................................   4         0      Mm2
    ..................................uuAcccaacuuucuaaaugagcug.....................................................   1         1      Mm2
    ...................................uAcccaacuuucuaaaugagc.......................................................   18        1      Mm2
    ...................................uAcccaacuuucuaaaugagcu......................................................   35        1      Mm2
    ...................................uAcccaacuuucuaaaugagcug.....................................................   136       1      Mm2
    ...................................uAcccaacuuucuaaaugagcugu....................................................   2         1      Mm2
    .....................................................................agacauuuggccUguuggguga....................   1         1      Mm2
    .....................................................................agacauuuggccaguuggguga....................   1         0      Mm2
    ......................................................................gacauuuggccaguuggg.......................   1         0      Mm2
    ......................................................................gacauuuggccaguugggug.....................   1         0      Mm2
    ......................................................................gacauuuggccaguugggugG....................   1         1      Mm2
    ......................................................................gacauuuggccaguuggguga....................   10        0      Mm2
    ......................................................................gacauuuggccaguugCguga....................   1         1      Mm2
    ......................................................................gacauuuggccaguugggugaU...................   2         1      Mm2
    ......................................................................gacauuuggccaguuggCugac...................   1         1      Mm2
    ......................................................................gacauuuggccaguugggugac...................   6         0      Mm2
    ......................................................................gacauuuggccaguugggugacu..................   1         0      Mm2

    ......................................................................Uacauuuggccaguuggguga....................   1         1      Mm1
    ......................................................................gacauuuggccaguuggguga....................   12        0      Mm1
    ......................................................................gacauuuggccaguugggugaU...................   4         1      Mm1
    ......................................................................gacauuuggccaguugggugac...................   5         0      Mm1
```
